# Pineal Abnormalities in Psychosis and Mood Disorders: A Systematic Review

**DOI:** 10.3390/brainsci13050827

**Published:** 2023-05-20

**Authors:** Satyam Chauhan, Andrei Barbanta, Ulrich Ettinger, Veena Kumari

**Affiliations:** 1 Department of Life Sciences, College of Health, Medicine and Life Sciences, Brunel University London, London UB8 3PH, UK; satyam.chauhan2@brunel.ac.uk (S.C.); andrei.barbanta@brunel.ac.uk (A.B.); 2 Department of Psychology, University of Bonn, 53111 Bonn, Germany; ulrich.ettinger@uni-bonn.de

**Keywords:** pineal gland, melatonin, MRI, schizophrenia, depression, bipolar disorder, biomarker

## Abstract

The pineal gland (PG) is a small interhemispheric brain structure that influences human physiology in many ways, most importantly via secretion of the hormone melatonin which is known to regulate sleep and wakefulness. Here, we systematically reviewed existing neuroimaging studies of PG structure, and/or melatonin release (MLT) in psychosis and mood disorders. Medline, PubMed, and Web of Science databases were searched (on 3 February 2023), yielding 36 studies (8 PG volume, 24 MLT). The findings showed smaller-than-normal PG volume in people with schizophrenia, regardless of symptom severity and illness stage; and smaller-than-normal PG volume in major depression, with some indication of this being present only in certain subgroups, or in those with high scores on the ‘loss of interest’ symptom. There was considerable evidence of lower-than-normal MLT as well as aberrant MLT secretion pattern in schizophrenia. A similar picture, though less consistent than that seen in schizophrenia, emerged in major depression and bipolar disorder, with some evidence of a transient lowering of MLT following the initiation of certain antidepressants in drug-withdrawn patients. Overall, PG and MLT aberrations appear to represent transdiagnostic biomarkers for psychosis and mood disorders, but further work is needed to establish their clinical correlates and treatment implications.

## 1. Introduction

For centuries, the pineal gland (PG) has been referred to as the ‘third eye’ or ‘ajna chakra’, ‘pineal eye’, and ‘seat of the soul’, and it was only in the late 19th century that we gained a clear understanding of its structure and various influences on the mammalian physiology, especially its role in the regulation of sleep cycle and wakefulness [1,2,3,4,5]. In humans, the PG is a small interhemispheric brain structure, resting proximally on the posterior aspect of the diencephalon. It is located within 1–2 mm of the midline and becomes visible between habenular and posterior commissures around 7 weeks of gestation [6]. Average PG dimensions in human adults are 5–9 mm in length, 1–5 mm in width, and 3–5 mm in height, and it weighs roughly between 100 and 180 mg depending on the age and sex [7,8]. The main function of the PG is to receive and transmit the light-dark signals from our surroundings and, accordingly, to produce and secrete the hormone melatonin [9]. Pineal melatonin (MLT) is known to modulate circadian rhythms and is involved in sleep regulation [10], reproductive physiology [8,11,12], and immunological regulation [13].

In recent decades, there has been much interest in examining PG and MLT production in people with psychosis and mood disorders, given that poor regulation and/or quality of sleep feature prominently in both of these disorders [14,15]. There are reports of smaller PG volumes, compared to healthy people, both in people with psychosis [16,17] and mood disorders [18] though not all studies have found this (e.g., [19]). It is possible that PG and MLT aberrations represent transdiagnostic biomarkers across these disorders or, alternatively, they might be associated with certain cross-diagnostic symptom dimensions. Of particular relevance in this context are depressive symptoms that not only characterise mood disorders but are also experienced by a significant proportion (25–70%) of people with schizophrenia [20,21] and may even precede clinical manifestation of psychosis [22,23]. Similarly, psychotic symptoms are experienced by subgroups of people with mood disorders [24]. To our knowledge, there is no published systematic review of PG and MLT aberrations across psychotic and mood disorders.

Our aim, therefore, was to systematically review, synthesise and appraise the findings of previous studies evaluating PG structure and/or MLT production in people with psychosis and/or mood disorders compared to healthy controls, and possible associations of PG structure/function with specific patient characteristics (symptom profiles, illness stage, treatment history, medication dose) within the psychosis and mood disorder groups to establish the extent to which specific PG abnormalities might represent disorder-specific or transdiagnosis effects and, in addition, be explained by illness-related influences (e.g., present in chronic but not first episode psychosis patients). We also consider any implications of our findings in psychosis and mood disorders for future research as well as the treatment and management of these disorders.

## 2. Methodology

This systematic review adhered to the Preferred Reporting Items for Systematic Reviews and Meta-Analyses (PRISMA) structure and guidelines [25].

### 2.1. Information Sources and Search

A literature search in Medline, PubMed, and Web of Science databases was conducted on 3 February 2023. The search terms included: (Pineal* OR melatonin OR “pineal gland”) AND (psychosis* OR psychot* OR schizophrenia*) AND (mood disorders* OR “affective disorders” OR “bipolar disorder” OR depress*). Search results were restricted to English, with no specific time window of publication. Cited references in the selected studies were also examined to identify further eligible literature.

### 2.2. Eligibility Criteria

Studies yielded by our literature search were assessed against the following inclusion criteria:

1. Study participants must have a diagnosis of schizophrenia/psychosis, bipolar disorder or major depression.
2. Allow comparison of the patient group/s with a health control group.
3. Report data on PG volume determined via magnetic resonance imaging (MRI) or MLT measured via blood, urine, saliva, or cerebrospinal fluid.
4. Studies must be primary research articles that have been peer-reviewed.

Studies without full text and methodology, meta-analyses, dissertation/PhD theses, unpublished papers, books, scoping, and systematic reviews were excluded. Animal studies, genetic and metabolic studies, post-mortem studies, and those with computerised tomography of PG were also excluded.

### 2.3. Study Selection

**Figure 1:**
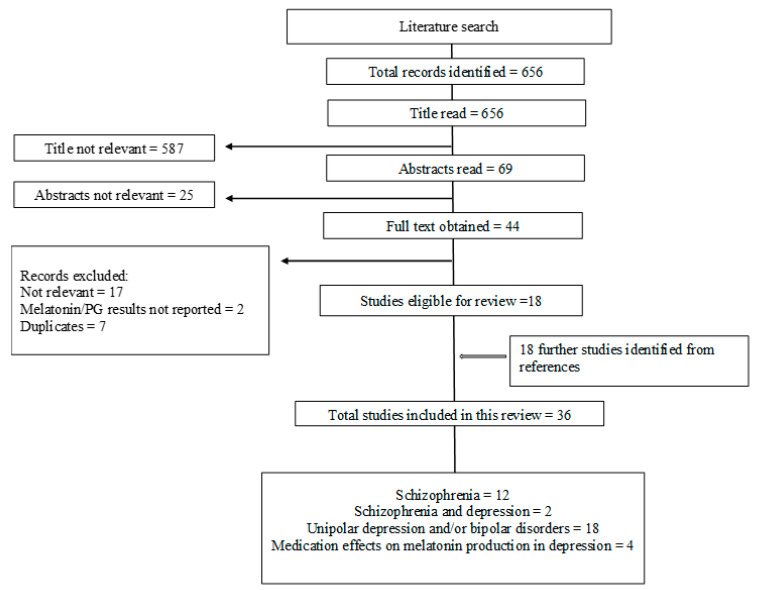
A flowchart of the study selection process following PRISMA guidelines.

All data resulting from the literature search were exported to Zotero. A screen of the results was conducted on a title and abstract basis for relevance and meeting the inclusion criteria by two independent reviewers (SC, AB). If the abstract did not contain sufficient information, the full text was retrieved before making a decision regarding the study meeting our inclusion/exclusion. The two reviewers (SC, AB) independently read the study title, abstracts, and full texts (where needed) and assigned each study a score of 0 (not relevant), 1 (questionable), or 2 (probably suitable). Next, the selection ratings of the two reviewers were compared, and the degree of agreement was assessed. Any discrepancies, as well as any studies with a score of 1, were discussed with a third reviewer (VK) to reach a consensus. The reasons for excluding studies at all stages were documented (for a flowchart of the study selection process, see Figure 1).

### 2.4. Data Collection Process

Data extraction for all selected studies was conducted by the first author (SC). In addition, data for a random selection of 18 studies were independently extracted by the second author (AB) to verify the extraction process.

### 2.5. Data Items and Analysis

For each of the selected study, the following data were extracted: author and study year, study population and sample characteristics (sample size, mean age and sex distribution for the patient and healthy comparison groups; and, in addition, for patient groups, diagnosis, age at illness onset, duration of illness, symptoms, current medication, and treatment history), design of the study, imaging modality or methods for assessing PG structure and/or function, intervention characteristics (where relevant), key study outcomes (i.e., diagnosis effects in PG structure and/or function; any association with patient characteristics), and funding sources. For all key outcome variables, group averages (mean, SD) and the size of the correlations (where reported between PG volume/MLT and relevant sample characteristics (e.g., symptoms or medication dose) were also extracted. All extracted data were compiled into a Microsoft Excel spreadsheet and analysed descriptively, considering the statistical significance of the findings as reported by the study authors for group comparisons (patients vs. healthy controls; or involving different patient groups) and/or correlations between the PG structure/function and patient characteristics. We made no specific assumptions where the study authors had assessed relevant sample characteristics (for example, symptoms) but not examined them in relation to the PG structure or function; we simply noted them as ‘not reported’ in the extracted data.

### 2.6. Quality Appraisal

The quality of selected studies was assessed using Newcastle-Ottawa Quality Assessment Scale [26] for non-randomised, cross-sectional studies, and Critical Appraisal Skills Programme [27] scores for randomised controlled trials (see Supplementary Tables S1 and S2 for quality rating of the selected studies).

## 3. Results

Overall, the search yielded 36 studies conducted in 16 different countries (UK, Germany, France, Sweden, Italy, Belgium, Poland, Canada, Brazil, USA, Australia, Turkey, Iran, China, Japan, and Taiwan). There was significant variability in sample sizes ranging between 5 and 87 participants for the healthy group, between 7 and 162 participants for the psychosis group (excluding bipolar disorder as this patient group was included mostly within the context of mood disorders, as bipolar versus unipolar depression), and between 6 and 50 participants for the mood disorders group (major depression or bipolar disorders). Eight of 36 reviewed studies investigated PG structure using MRI (4 studies in psychosis or schizophrenia, 3 studies in mood disorders, and 1 study involved schizophrenia as well as mood disorders), and 24 studies examined MLT in blood, urine and/or cerebrospinal fluid (8 in psychosis, 15 in mood disorders, 1 study involved both schizophrenia and mood disorders). Most of these 32 studies used a cross-sectional study design comparing the patient (psychosis and/or mood disorders) and healthy control groups, and some studies also examined possible associations between PG structure and/or MLT and symptom ratings or medication within the patient samples. Lastly, four studies examined medication effects on MLT in major depression and also included comparison with healthy controls. No study met our eligibility criteria for examining antipsychotic effects on MLT.

### 3.1. PG Volumes: Effects of Diagnosis and Possible Association with Clinical Characteristics

The details and key findings extracted from the reviewed studies in relation to PG volumes in psychosis and mood disorders are presented in Table 1.

PG volume was found to be significantly smaller in people with schizophrenia or psychosis, compared to healthy controls, in four of the five studies that investigated this [16,17,28,29]. An early study with a modest sample size [19] reported no significant difference between the patient and control groups although the mean PG volume was still numerically lower in the patient group (see Table 1a,b). In the two most recent studies with relatively large sample sizes [17,29], PG volume was found to be smaller-than-normal not only in the first-episode and chronic schizophrenia groups, but also in those at a high risk of developing psychosis. Furthermore, no relationship between the PG volume and age or any clinical characteristics of the patient sample (symptoms, age of illness onset, duration of illness and/or treatment) was detected in any of the four studies that found significantly smaller-than-normal PG volume in psychosis or schizophrenia samples [16,17,28,29].

PG volume was also found to be significantly smaller in people with major depressive disorders, compared to healthy controls, in two of the three studies that examined this [16,18]; however, there was no association between PG volumes and depressive symptom ratings in either of these studies (see Table 1b,c). One study [30] that did not observe a significant difference between the PG volumes of currently depressed or remitted groups of mood disorder patients and healthy controls, however, reported significantly smaller PG volumes in non-melancholic patients, compared to melancholic depressed patients; it also reported a negative association between PG volumes, and the ‘loss of interest’ symptom ratings but no association with any other symptoms (for example, the Beck Depression Inventory-II scores) when examined across the patient sample. Furthermore, this study [30] showed no significant difference between the PG volumes of bipolar disorder patients and healthy controls, and no significant association between total PG volume and any clinical characteristics, namely, the number of episodes, duration of illness or the medication dose, in this group of patients. A further study of bipolar disorder patients [31] also found them to not differ from healthy controls in PG volumes, although a group of BD patients were found to have smaller PG volumes than the controls in a later study [16].

There was only one study [16] directly comparing PG volumes in schizophrenia and mood disorders, and it showed (i) significantly lower PG volume in the schizophrenia group, compared to patient groups with unipolar depression or bipolar disorder, (ii) no significant difference between unipolar depression and bipolar disorder groups; (iii) and significantly lower PG volumes, compared to controls, in both unipolar depression and bipolar disorder groups (as already noted, both unipolar depression and bipolar disorder groups had larger volumes compared to those in people with schizophrenia).

**Table 1:** Studies examining pineal gland (PG) volume in patients with psychosis or mood disorders, compared to healthy controls.

Author and Year (in Chronological Order)	Patients (*n*, Diagnosis, Sex and Age)	Healthy Comparison Group (*n*, Sex and age)	Patient Characteristics			Imaging Details	Study Design	Key Outcomes			Study Quality Assessment (NOS Total) Scores
			Age at Illness Onset (AIO) and Duration of Illness (DoI)	Symptom Rating Measure/s (Mean ± SD)	Treatment History and Medication Duration			Group Differences	Direction of Group Effects	Association with Patient Characteristics	
**a. PG volume in patients with psychosis, compared to healthy controls**											
Rajarethinam et al. (1985) [19]	45 SZ or psychosis (31 M, 14 F; mean age: 30.2 ± 9.7)	86 (44 M, 42 F; mean age (27.3 ± 9.6)	Not reported	Not reported	Not reported	MRI with 1.5 GE tesla scanner	Between-groups	No significant difference in PG volume between patients (mean ± SD:0.208 ± 0.099 cm^3^) and controls (0.213 ± 0.097 cm^3^)	=	Not reported	4
Bersani et al. (2002) [28]	15 SZ (all M; mean age: 26.60 ± 5.28)	16 controls (all M; mean age: 29.26 ± 6.26)	AIO 18.42 ± 4.78 years; DoI 8.28 ± 4.59 years	SAPS: 55.66 ± 22.84; SANS: 62.17 ± 28.58; PANSS: 106.4 ± 22.93	Mean neuroleptic treatment duration (in years): 5.59 ± 5.40	MRI with 1.5 magnetom-siemens	Between-groups	Significantly smaller PG volume in patients (64.05 ± 20.69 mm^3^) than controls (74.62 ± 33.53 mm^3^).	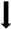	No correlation between PG volumes and age of clinical characteristics (AOI, DoI, symptoms)	6
Takahashi et al. (2019) [29]	At baseline 64 first-episode psychosis (37 M, 27 F; mean age: 24.0 ± 4.7) 40 chronic SZ (20 M, 20 F; mean age: 29.0 ± 5.5) 22 ARMS (11 M, 11 F; mean age: 19.1 ± 4.1)	At baseline 86 controls (48 M, 38 F; mean age: 23.7 ± 5.4)	At baseline First-episode: AIO 23.1 ± 4.7 years; DoI 11.2 ± 12.2 months; Chronic SZ: AIO 20.9 ± 4.4 years; DoI 96.8 ± 39.8 months	At baseline First-episode: SAPS: 27.3 ± 21.9; SANS: 53.1 ± 25.2 Chronic SZ: SAPS: 30 ± 19.2; SANS: 45.5 ± 18.7; ARMS: SAPS: 20.4 ± 10.9; SANS: 48.5 ± 19.4 Also, BDI: 24.1 ± 10.00; STAI trait: 65.3 ± 10.9; STAI state: 58.4 ± 11.3	At baseline Mean (± SD) medication duration (in months) for ARMS (2.3 ± 4.1); First-episode (8.3 ± 12.6); Chronic SZ (72.4 ± 47.6) Medication type: ARMS (1 typical, 3 atypical); first-episode (18 typical, 43 atypical, 1 mixed); chronic SZ (19 typical, 18 atypical, 3 mixed)	MRI with 1.5 tesla scanner	Between-groups (with a follow-up)	At baseline Significantly smaller PG volumes in ARMS (102.7 ± 43.2 mm^3^), first-episode (102.3 ± 46.2 mm^3^), and chronic SZ patients (105.7 ± 46.4 mm^3^) compared to healthy controls (131.1 ± 60.1 mm^3^).	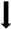	PG volumes not correlated with demographic (age or education level) or clinical variables (AIO, DoI, duration of treatment, symptoms).	7
	At follow-up (after 15.6 ± 17.4 months) 23 first-episode psychosis (15 M, 8 F; mean age: 23.5 ± 4.8) 16 chronic SZ (7 M, 9 F; mean age: 31.6 ± 7.1) 22.7% ARMS developed SZ	At follow-up 21 controls (13 M, 8 F; mean age: 24.5 ± 5.0)		At follow-up First-episode: SAPS: 17.0 ± 17.1; SANS: 38.0 ± 22.5 Chronic SZ: SAPS: 34.9 ± 30.0; SANS: 57.3 ± 18.7	At follow-up Medication type: First-episode (3 typical, 16 atypical, 4 mixed); chronic SZ (4 typical, 8 atypical, 4 mixed)			At follow-up No effect of time, diagnosis or any interaction.	=		
Takahashi et al. (2022) [17]	162 first-episode patients (108 M, 54 F; mean age: 21.5 ± 3.4) 89 chronic SZ (76 M, 13 F; mean age: 34.9 ± 9.6) 135 clinical high risk (78 M, 57 F; mean age: 20.1 ± 3.6)	87 controls (55 M, 32 F; mean age: 26.9 ± 10.1)	First-episode: AIO not reported; DoI 54 ± 87 (days) Chronic SZ: AIO not reported; DoI 4673 ± 3613 (days)	Clinical high risk: BPRS total: 44.1 ± 8.3; BPRS psychotic subscale: 8.4 ± 2.6; SANS 18.9 ± 12.7	Mean antipsychotic duration (chlorpromazine equivalent) First-episode: 154.7 ± 118.2 mg/day Chronic SZ: 842.9 ± 715.8 mg/day	MRI with 1.5 tesla scanner	Between-groups	Significantly smaller PG volume in CHR (97.5 ± 50.3 mm^3^), FEP (97.7 ± 51.2 mm^3^), CSz (99.0 ± 61.5 mm^3^) groups than controls (139.4 ± 73.9 mm^3^). Pineal parenchymal volume smaller in all clinical groups (CHR: 93.4 ± 42.3 mm^3^; FEP:94.2 ± 43.5 mm^3^; CSz: 96.8 ± 58.8 mm^3^) than controls (132.9 ± 62.7 mm^3^, with no significant difference between the clinical groups.	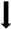	PG and parenchyma volumes not correlated with age, DoI or antipsychotic dose in the first-episode or chronic SZ groups; and not correlated with symptoms in the high-risk group.	6
**b. PG volume in patients with schizophrenia, compared to those with major depression or bipolar disorders and healthy controls**											
Findikli et al. (2015) [16]	17 SZ (11 M, 6 F; mean age: 36.6 ± 12.7)	30 controls (16 M, 14 F; mean age: 41.1 ± 13.3)	AIO 29.00 ± 12.02 years; DoI 7.8 ± 6.2 years	Not reported	Mean antipsychotic treatment duration 7.1 ± 6.5 years (risperidone, amisulpiride, flupentixol)	MRI with 1.5 Tesla	Between-groups	Significantly smaller PG volumes in SZ patients (83.5 ± 10.1 mm^3^) had than controls (99.7 ± 12.03 mm^3^). Smaller PG volume in SZ (83.5 ± 10.1 mm^3^) compared to BD (93.7 ± 11.4 mm^3^) and UD groups (95.1 ± 11.2 mm^3^) (see below).	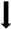	PG volumes not correlated with clinical characteristics (AIO, DoI, treatment duration).	7
	16 UD (8 M, 8 F; mean age: 39.4 ± 13.9) 17 BD (11 M, 6 F; mean age: 30 ± 10.2)	30 controls (16 M, 14 F; mean age: 41.1 ± 13.3)	UD: AIO 35.3 ± 15.5 years; DoI 4.1 ± 4.2 BD: AIO 25.06 ± 10.8 years; DoI 4.3 ± 4.09 years	Not reported	All UD patients on SSRIs Most BD patients on antipsychotics and 2 received lithium treatment.			Smaller PG volume in both UD (95.1 ± 11.2 mm^3^) and BD patients (93.7 ± 11.4 mm^3^) compared to controls (99.7 ± 12.03 mm^3^).	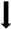		
**c. PG volume in patients with unipolar depression or bipolar disorder, compared to healthy controls**											
Sarrazin et al. (2012) [31]	16 BD I and II (10 M, 6 F; mean age: 42.8 ± 8.3)	16 controls (8 M, 8 F; mean age: 37.1 ± 11.35)	Not reported	Not reported	15 patients medicated	3-T MRI magnet	Between-groups	No difference in PG volume between patients (115.3 ± 54.3 mm^3^) and controls (110.4 ± 40.5 mm^3^).	=	Not reported	5
Zhao et al. (2019) [18]	50 MDD (19 M, 31 F; mean age: 42.10 ± 10.52)	35 controls (18 M, 17 F; mean age: 42.74 ± 10.04	Not reported	Patients: HDRS: 29.42 ± 11.59; PSQI: 11.96 ± 5.43; HAMA: 19.06 ± 7.41 Controls: HDRS: 2.49 ± 2.37; HAMA: 2.86 ± 3.72	29 patients on SSRIs (paroxetine, escitalopram, fluvoxamine), 1 on NaSSA (mirtazapine), and 20 on SNRIs (venlafaxine. Duloxetine, sertraline)	MRI 3.0 Tesla MR system	Between-groups	Significantly smaller pineal parenchymal volume in MDD (70.47 ± 28.30 mm^3^) relative to controls (89.87 ± 38.81 mm^3^).	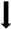	No correlation between pineal parenchymal volume and symptoms (HAMD, HAMA, and PSQI).	7
Takahashi et al. (2020) [30]	29 cMDD (7 M, 22 F; mean age: 32.5 ± 8.3) 27 rMDD (9 M, 18 F; mean age: 35.1 ± 10.0)	33 controls (12 M, 21 F; mean age: 34.0 ± 9.9)	cMDD: AIO 21.1 ± 8 years; DoI not reported rMDD: AIO 26.0 ± 9.4 years; DoI not reported	cMDD: BDI: 36.8 ± 8.9; PANAS positive affect: 21.6 ± 6.5, PANAS negative affect: 21.2 ± 8.5; MASQ general depression: 47.3 ± 9.2 rMDD: BDI: 13.0 ± 11.7; PANAS positive affect: 28.7 ± 8.0; PANAS negative affect: 14.2 ± 4.7; MASQ general depression: 35.0 ± 11.7 Healthy controls: BDI: 3.6 ± 4.1; PANAS positive affect: 32.9 ± 7.3; PANAS negative affect 11.2 ± 1.6; MASQ general depression: 19.5 ± 7.2	MDD- 21 current and 12 remitted MDD patients took medication in the past 6 months.	MDD- MRI using 1.5 Tesla siemens scanner	Between-groups	No significant difference MDD and control groups: current MDD, PG: 119.2 ± 51.5 mm^3^; parenchymal: 115.0 ± 45.1 mm^3^; remitted MDD, PG: 119.7 ± 53.7 mm^3^; parenchymal: 116.5 ± 48.2 mm^3^; controls, (PG: 145 ± 84.9 mm^3^; parenchymal: 138.8 ± 71.7 mm^3^ Significantly smaller PG volume, however, in the non-melancholic subgroup of MDD (*n*:19, parenchymal: 101.9 ± 42.4 mm^3^) than the melancholic MDD subgroup (*n*:10; parenchymal: 142.0 ± 40.7 mm^3^).	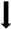 =	Total pineal and parenchymal volume in cMDD patients correlated negatively (r = −0.571) with MASQ loss of interest score (but no correlation with number of episodes, and other MASQ, PANAS, and BDI scores).	8
	26 BD (8 M, 18 F; mean age: 38.4 ± 10.9) Note: 16 of 28 BD had psychotic symptoms.	24 controls (7 M, 17 F; mean age: 38.7 ± 11.1) (a subsample of the controls noted above)	BD: AIO 13.5 ± 10.1 years; DoI not reported	Not administered	12 BD patients on lithium (dosage, (975 ± 213) and 12 on valproate (1437 ± 594).	BD- MRI using 1.5 Tesla GE sigma scanner		No significant difference between BD (PG: 121 ± 79.0 mm^3^; parenchymal: 119.6 ± 76.8 mm^3^) and controls (PG: 129.8 ± 62.0 mm^3^; parenchymal: 126.4 ± 57.6 mm^3^).	=	No correlation between pineal/parenchymal volumes and number of episodes, medication dose, or DoI in patients.	

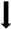
 denotes significantly lower volume in patient group/s compared to the comparison group; = denotes no difference between the patient and the comparison group. Abbreviations: AIO, Age at Illness Onset; ARMS, At Risk Mental State; BDI, Beck Depression Inventory [32]; BPRS, Brief Psychiatric Rating Scale [33]; CAARMS, Comprehensive Assessment of at-Risk Mental State [34]; CHR, Clinical High Risk; cMDD, Current Major Depressive Disorder; DoI, Duration of Illness; F, Females; FEP, First Episode Psychosis; HAMA, Hamilton Rating Scale for Anxiety [35]; HDRS, Hamilton Depression Rating Scale [36]; M, Males; MASQ, Mood and Anxiety Symptom Questionnaire [37]; MDD, Major Depressive Disorder; MRI, Magnetic Resonance Imaging; MRI, Magnetic Resonance Imaging; NaSSA, Noradrenergic and Specific Serotonergic Antidepressants; NOS: Newcastle-Ottawa Quality Assessment Scale [26]; PANAS, Positive and Negative Affect Scale [38]; PANSS, Positive and Negative Syndrome Scale [39]; PG, Pineal Gland; PSQI, Pittsburgh Sleep Quality Index [40]; rMDD, Remitted Major Depressive Disorder; SANS, Scale for the Assessment of Negative Symptoms [41]; SAPS, Scale for the Assessment of Positive Symptoms [42]; SNRIs, Serotonin-Norepinephrine Reuptake Inhibitors; SSRIs, Selective Serotonin Reuptake Inhibitors; STAI, State-Trait Anxiety Inventory [43]; SZ, Schizophrenia, UD, Unipolar Disorder; BD, Bipolar Disorder.

### 3.2. PG Function (MLT): Effects of Diagnosis and Association with Clinical Characteristics

There were eight studies examining MLT level or MLT secretion patterns in people with schizophrenia (see Table 2). All these studies, except one [44], observed (overall) lower-than-normal MLT level [45,46,47,48] or an aberrant pattern of MLT secretion in this group of patients [48,49,50]. Specifically, there was a different pattern in patients and controls, with MLT increasing at 8 p.m. and peaking at 2 a.m. in controls but no significant peaks in patients [49]; earlier peaks in drug-free patients, relative to controls [49]; and delayed rhythm and atypical sleep-wake cycles in 50% of the patients, relative to controls [50]. In one study [48] that compared acutely unwell and chronic patient groups, acutely unwell patients had higher MLT, relative to chronic patients, but both groups still had lower MLT relative to healthy controls.

There was no relationship between MLT aberrations and symptoms in most of the studies that examined this in people with schizophrenia [45,47,49], and in one study [50] that did observe lower MLT in patients with positive symptoms, relative to those without positive symptoms, this effect was explained by age (Table 2). Antipsychotic medication seemed to be associated with a change towards normalisation of the MLT secretion pattern in that medicated patients did not differ significantly from controls [51]. In the only study [44] that did not find lower-than-normal MLT levels in schizophrenia patients, MLT levels were found to be positively associated with antipsychotic medication dose as well greater symptom severity, possibly reflecting higher antipsychotic doses prescribed to those with greater symptom severity.

There were 15 studies examining MLT level or MLT secretion in people with major depression and/or bipolar disorders (Table 3). Most of these studies showed significantly lower MLT, especially nocturnal MLT, in depressed patients, compared to healthy controls [52,53,54,55,56,57,58], or atypical MLT secretion patterns such as delayed peak [59] or delayed onset [60]. Relatively fewer studies showed a non-significantly lower MLT in patients [61], no significant difference between the patient and control groups [62,63], or lower MLT level in patients with psychotic depression, but higher in those with non-psychotic depression, both compared to controls [64]. One of these studies [56] had also examined and observed lower MLT in panic disorder patients, compared to healthy controls. Interestingly, in one study [65] that had assessed MLT in both blood and cerebrospinal fluid (CSF), blood but not CSF MLT was lower in depressed patients, and CSF but not blood MLT was lower in bipolar disorder patients, both relative to healthy controls. However, an earlier study [66] had shown lower serum MLT in bipolar disorder patients (at 1 a.m. and 5 a.m.; but only at 1 a.m. in euthymic subgroup) relative to the healthy group.

Most studies in mood disorders had only small-to-modest and mixed sex samples (with marked inter-study variation in average depression severity, see symptom ratings for individual studies, Table 3), and did not examine relationship with symptom profiles or medication. A few studies which did examine such associations presented a mixed picture, with no relationships between basal MLT or secretion patterns and medication [52] or symptom severity [55], or a negative relationship with depression severity [53]. Some studies also showed MLT to correlate positively with body mass index (BMI), and to be influenced by sex of the participant while others did not (Table 3). Lastly, there was only one study [67] comparing MLT in people with schizophrenia (*n* = 12) with that in people with depression (*n* = 60] and, unexpectedly, its findings revealed higher MLT in schizophrenia, relative to depressed patients (Table 4).

There were four studies (all involving 20 or fewer patients) investigating the influence of antidepressant medications on MLT in depressed patients (Table 5). Two of these studies, with only or predominantly females, showed a transient lowering of MLT following certain antidepressants [desipramine [68]; fluoxetine [69]], while the remaining two mixed-sex studies showed no pre- to post-change in MLT [clomipramine [70]; fluoxetine [71]]. Further complicating the picture regarding MLT patterns in depressed patients, three of these four studies [68,69,71] had reported no significant differences in MLT between the patient and control groups at baseline, while one study [70] reported higher MLT in patients, relative to healthy controls (see Table 5).

**Table 2:** Studies examining melatonin production (MLT) in people with psychosis, compared to healthy controls.

Author & Year (in Chronological Order)	Patients (*n*, Diagnosis, Sex and Age)	Healthy Comparison Group (*n*, Sex and Age)	Patient Characteristics			Technique Used to Measure MLT	Study Design	Key Outcomes			Study Quality Assessment (NOS Total) Scores
			Age at Illness Onset (AIO) and Duration of Illness (DoI)	Symptom Rating Measure/s (mean ± SD)	Treatment History and Medication Duration			Group Differences	Direction of Group Effects	Association with Patient Characteristics	
Ferrier et al. (1982) [45]	21 chronic SZ (all M; mean age: 59.6 ± 8.4)	12 controls (all M; mean age: 54.2 ± 9.1)	Not reported	MIRS (data not reported)	All patients drug free for at least 1 year	MLT (at mid-night and 8 a.m.) measured via blood	Between-groups	Significantly lower MLT level in patients (16.2 ± 12.8 mL) than controls (31.9 ± 17.6 mL) at mid-night; and non-significantly lower (9.7 ± 3.2 mL) at 8:00 a.m. than controls (10.8 ± 5.0 mL)	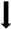	MLT did not correlate with age, DoI or symptom rating. A significant positive relationship between MLT and BMI in both groups (controls: r = 0.66; patients r = 0.35).	7
Beckman et al. (1984) [44]	28 paranoid SZ (all M; mean age: 40.6 ± 8.0)	16 controls (all M; mean age: not reported)	Not reported	BPRS (mean or range not reported)	15 patients treated with butyrophenones and phenothiazines for at least 3 weeks (mean dose: 585 ± 755 mg/day). Remaining 13 patients not on any drug for at least 4 weeks. Prior treatment history not reported.	MLT (between 9–10 a.m.) analysed in the CSF	Between-groups	No significant differences in MLT between the patients with (15 ± 16 mL) or without neuroleptic medication (15 ± 14 mL) and controls (15 ± 25 mL). No difference between medicated and drug free patients.	=	MLT positively correlated with symptom severity (r = 0.34) and antipsychotic dose (r = 0.47).	6
Fanget et al. (1989) [46]	23 SZ (10 M, 13 F; mean age: 41.5 ± 11.6)	26 controls (16 M, 10 F; mean age: 40.7 ± 4.4)	AIO not reported DoI 18.4 ± 9.5 (years)	BPRS: 60.3 ± 15	15 patients on levopromazine, 8 on haloperidol, 21 on anticholinergic drug, 10 on benzodiazepines, and 4 on antidepressants Mean treatment duration: 16 years	MLT concentration (at mid-night) determined via blood radioimmunoassay	Between-groups	Significantly lower MLT in patients (52.3 ± 41.8 mL) compared to controls (75.5 ± 18.7 mL) but significant differences between drug-free patients and those on antidepressants.	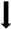	Not reported	5
Monteleone et al. (1992) [49]	7 paranoid SZ (all M; mean age: 29.1 ± 3.5)	7 controls (all M; mean age: 30.8 ± 3.3)	AIO not reported DoI 8.8 ± 3.3 (years)	BPRS: 41.1 ± 4.7; HDRS: 6.8 ± 3.7	5 patients drug free for 3 weeks and the remaining 2 for 1 year. Medication doses/type and mean age of medication duration not reported.	MLT concentration (over a 24 h period) determined via plasma using RIA method	Between-groups	Different MLT secretion pattern in patients and controls; MLT increasing at 8 p.m. and peaking at 2 a.m. in controls but no significant peaks observed in patients.	*~*	MLT not correlated with HDRS and BPRS ratings.	7
Rao et al. (1994) [51]	89 drug-free SZ or schizoaffective disorder (47 M, 42 F; mean age: M, 34 ± 12; F, 36 ± 11) Medicated patients (11 M, 14 F; mean age: M, 26 ± 8; F, 41 ± 14)	34 controls (17 M, 17 F; mean age: M, 24 ± 4; F, 24 ± 2)	AIO not reported DoI > 6 months in all patients	Not reported	25 patients on a stable dose of neuroleptic medication for 5 years, 21 patients were drug free and the remaining 68 were on a minimum of 3 day wash out period. Medication doses/type and mean age of medication duration not reported.	MLT (over a 24 h period) measured via blood	Between-groups	Different MLT secretion pattern in patients and controls; MLT peaked significantly earlier in drug-free patients and similar but non-significant phase advancement in medicated SZ patients, both relative to controls.	*~*	Not reported	5
Jiang & Wang (1998) [47]	21 paranoid SZ (all M; mean age: 27.3 ± 7.2)	21 controls (all M; mean age: 29.7 ± 11.0)	AIO not reported DoI 0.6–12 months (3.12 ± 2.43)	Patients: BPRS: 48.3 ± 5.8; HDRS: 7.6 ± 3.6 Controls: BPRS: 6.1 ± 4.2; HDRS: 4.8 ± 3.7	All patients on antipsychotic medications (chlorpromazine equivalent 600–1200 mg/day; mean not reported)	MLT (over 24 h period) measured via blood		Significantly lower MLT in patients (38.1 ± 22.5 ng/mL) than patients (22.2 ± 11.6 ng/mL).	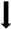	MLT did not correlate with age, BMI, DoI or symptom severity. Relationship with medication not reported.	7
Vigano et al. (2001) [48]	13 SZ (6 M, 7 F; median age: 26; range: 20–37)	20 controls (sex distribution or age not reported)	Not reported	Not reported	Eight patients drug free and 5 on neuroleptic drugs Medication doses/type and mean age of medication duration not reported.	MLT (over a 24 h period) measured via blood	Between-groups	Both daytime and nocturnal MLT levels significantly lower in patients than controls (mean values not reported).	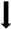	Not reported	6
Wulff et al. (2012) [50]	20 paranoid SZ (15 M, 5 F; mean age: 38.8 ± 8.6)	21 controls (13 M, 8 F; mean age: 37.5 ± 9.6)	AIO not reported DoI 2–33 years (median = 10)	Patients: PSQI: 8.32 ± 3.77; POMS week 1: 13.3 ± 10.7; POMS week 6: 14.3 ± 10; MEQ: EC = 72.3%, IC = 27.8%, MC = 0% Controls: PSQI: 4.83 ± 2.37; POMS week 1: 12.1 ± 8.4; POMS week 6: 9.5 ± 9; MEQ: EC = 65%, IC = 30%, MC = 5%	12 patients on antipsychotics only, 7 received additional psychotropic medication	MLT sulphate concentrations (over a 24 h period) measured in urine by radioimmunoassay	Between-groups	50% of the patient sample reported significant delayed MLT rhythms and free running sleep-wake cycles relative to controls.	*~*	Lower MLT in patients with (MLT: 467 ± 228.6 ng) than those without positive symptoms (MLT: 1066.3 ± 501 ng). Patients had poor sleep quality than controls but did not differ for chronotype, mood profile or age. MLT did not correlate with antipsychotic medication.	8

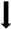
 denotes significantly lower MLT in patients compared to the healthy controls; ~ denotes altered MLT secretion pattern in patients relative to healthy controls; = denotes no difference between the patient and the healthy control group in MLT level or secretion pattern. Abbreviations: AIO: Age at Illness; BPRS, Brief Psychiatric Rating Scale [33]; CSF, Cerebrospinal Fluid; EC, Evening Chronotype; F, Females; HDRS, Hamilton Depression Rating Scale [36]; IC, Intermediate Chronotype; MDD, Major Depressive Disorder; M, Males; MC, Morning Chronotype; MEQ, Morningness-Eveningness Questionnaire [72]; MG, Milligram; MIRS, Modified Inpatient Rating Scale [73]; MLT, Melatonin; POMS, Profile of Mood States [74]; PSQI, Pittsburgh Sleep Quality Index [40].

**Table 3:** Studies examining MLT production in people with depressive and/or bipolar disorders, compared to controls.

Author & Year (in Chronological Order)	Patients (*n*, Diagnosis, Sex and Age)	Healthy Comparison Group (*n*, Sex and Age)	Patient Characteristics			Technique Used to Measure MLT	Study Design	Key Outcomes			Study Quality Assessment (NOS Total) Score
			Age at Illness Onset (AIO) and Duration of Illness (DoI)	Symptom Rating Measure/s (Mean ± SD)	Treatment History and Medication Duration			Group Differences	Direction of Group Effects	Association with Patient Characteristics	
Beck-Friis et al. (1984) [52]	30 acutely ill MDD inpatients (13 M, 17 F; mean age: 44 ± 1.9) 24 chronic outpatients with UD (6 M, 6 F) or bipolar disorder (6 M, 6 F; mean age: 51 ± 2.1)	33 controls (14 males, 19 females; mean age: 40 ± 2.2)	Not reported	MDD: CPRS:2 ± 0.1 Chronic UD and BD: CPRS:0.3 ± 0.1 Controls: CPRS:0.0 ± 0.0	All patients on medication (a range of medications used)	Serum MLT (over a 24 h period) measured via blood.	Between-groups	Significantly lower nocturnal MLT in both patient groups (acute MDD: 0.25 ± 0.03 nmol/L; chronic UD or BD 0.17 ± 0.02 nmol/L) than controls (0.30 ± 0.03 nmol/L).	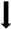	MLT negatively correlated with BMI (r = −0.45) but no association with medication.	5
Claustrat et al. (1984) [54]	11 affective disorder (1 M, 10 F; mean age: 44.5 ± 11.1)	24 controls, including 8 young M (mean age: 27.3 ± 3.1), 8 older M (62 ± 4.2), and 8 young F (24.5 ± 5.1)	Not reported	HDRS: 41.90 ± 6.64	Drug free for at least 10 days	MLT (over a 24 h period) measured via blood	Between-groups	Significantly lower in patients (22.5 ± 19.2 mL) than controls (41.2 ± 9.5 mL). MLT level peaked at 3 a.m. in controls (young males: 86.5 ± 22 mL; ovulating women: 72.9 ± 26 mL; older males = 84.7 ± 20 mL) but decreased in patients (39.3 ± 38.4 mL).	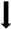	Not reported	6
Brown et al. (1985) [55]	Sample 1 from Philadelphia: 7 depressed melancholia patients (all M; mean age: 42 ± 4)	Sample 1 from Philadelphia: 5 controls (all M; mean age: 32 ± 4)	Not reported	Patients: HDRS: 31 ± 3.4	All patients drug free for at least 7 days, and did not receive electroconvulsive therapy within 3 months of testing.	MLT measured (over 15 h period) via blood	Between-groups	Patients had lower MLT (53 ± 10 mL) than the controls (89 ± 9 mL).	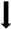	MLT levels did not correlate with age or symptom severity.	6
	Sample 2 from New York: 19 depressed melancholia (5 M, 14 F; mean age: 62.4 ± 2.5) 9 depressed non-melancholia (all F; mean age: 57.3 ± 4.5)	Sample 2 from New York 7 controls (all M; mean age: 41.4 ± 4)		Depressed melancholia: HDRS: 35.9 ± 4.3 Depressed non-melancholia: HDRS: 23.1 ± 1.7		MLT measured (at 9 a.m. and 11 p.m.) via blood		Significantly lowered MLT at 11:00 p.m. in melancholic (36.4 ± 4.6 mL) and non-melancholic (58.6 ± 9.6 mL) groups relative to controls (60.3 ± 8.0 mL); at 9 p.m., no significant group differences.		MLT levels did not correlate with age or symptom severity.	
Beck-Friis et al. (1985) [53]	32 MDD (14 M, 18 F; mean age: 43 ± 1.9)	33 controls (14 M, 19 F; mean age: 40 ± 2.2)	Not reported	CPRS (mean values not reported)	Not reported	MLT (over 24 h period) measured via blood	Between- groups	Lower in MLT level in patients with abnormal DST (0.19 ± 0.03 nmol/L) but not normal DST patients: (0.30 ± 0.02 nmol/L) relative to controls (0.30 ± 0.03 nmol/L). MLT significantly lower in patients depressed for >3 summer periods (0.17 ± 0.04 mL) compared to those depressed for <3 summer periods. (0.28 ± 0.04 mL).	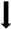	No relationship found between MLT and DoI or diagnostic subcategories. Negative associations of MLT with CPRS global score, depressed mood, and retardation symptoms (r values not reported). MLT relationship with demographics and medication not reported.	6
Thompson et al. (1988) [62]	9 depressive patients (4 M, 5 F; mean age: 48.1; age range: 26–66; SD not reported)	9 controls (4 M, 5 F; mean age: 46.1; age range: 28–58; SD not reported)	Not reported	CRG:7.3 (range: 3–10); SD not reported HDRS:25.1 (range: 10–31); SD not reported	Patients antidepressant-free for at least 6 weeks but allowed benzodiazepines (exact numbers and medication dose not reported)	MLT levels (over 24 h period) measured via blood	Between- groups	No significant difference between patients and controls (mean values not reported).	=	Not reported	5
McIntyre et al. (1989) [56]	11 MDD-melancholic subtype (4 M, 7 F; mean age: 58 ± 5.0) 13 panic disorder (1 M, 12 F; mean age: 36 ± 2.1)	18 controls (4 M, 14 F; mean age: 32 ± 2.2)	Not reported	MDD melancholic: HDRS: 21.2 ± 2.1 Panic disorder: HDRS: < 8	Most MDD patients on antidepressants, 4 drug free at least 5 days. 8 panic disorder patients receiving benzodiazepines, 4 received additional tricyclic antidepressants and 1 drug free. Medication dose not reported.	MLT (between 8 p.m. and midnight) measured via blood	Between-groups	Significantly lower MLT in both MDD (27.1 ± 5.1 mL) and panic disorder (28.4 ± 6.4 mL) groups compared to the controls (51.6 ± 4.1 mL).	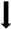	Not reported	5
Kennedy et al. (1996) [66]	9 BD (2 M, 7 F; mean age: 33.3 ± 15.5)	12 controls (sex distribution not reported; mean age: 25.3 ± 4.9)	Not reported	Not reported	Patients medication free for least 2 weeks	MLT (between 8 p.m. and 6 a.m.) measured via blood and (over 24 h period) via urine samples.	Between-groups	Lower serum MLT in BD patients than controls at 1 a.m. and 5 a.m. (though euthymic patients had significantly lower MLT levels at 1 a.m. only). Interestingly, no significant difference between the patient and control groups in daytime or nocturnal urinary MLT.	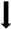	Not reported	5
Shafii et al. (1996) [64]	22 children (9 M, 13 F; age range: 8 to 17) of whom 18 had MDD, 1 dysthymia, and 3 with BD.	19 controls (11 M, 8 F; mean age/range not reported:)	Not reported	Children Depression Inventory, Children Depression Rating Scale (mean values not reported)	Two weeks of drug-naïve or drug-free prior to the study.	MLT (between 6 p.m. and 7 a.m.) measured via blood	Between-groups	Significantly higher nocturnal MLT levels in patients (0.18 ± 0.14 nmol/L) compared to controls (0.15 ± 0.10 nmol/L). When the MDD group was split into non-psychotic (*n* = 15) and psychotic (*n* = 7) sub-groups, lower MLT in the psychotic subgroup (0.11 ± 0.11 nmol/L), but higher MLT in the non-psychotic subgroup (0.19 ± 0.15 nmol/L) relative to controls.	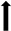	MLT positively correlated with height (r = 0.09) and weight (r = 0.17) but relationship with symptom ratings, and other variables not reported.	5
Voderholzer et al. (1997) [61]	9 MDD (6 M, 3 F; mean age: 29 ± 7)	9 controls (sex not reported; mean age: 28 ± 7)	AIO not reported DoI 3.9 ± 3.7 (months) Range 2 weeks–10 months	HDRS: 32 ± 6	5 patients drug free and 4 patients who took psychotropic medication underwent a washout period of at least 3 weeks.	MLT (over 24 h period) measured via blood.	Between-groups	Slightly but not significantly lower 24 h and nocturnal MLT in patients (24 h: 33 ± 19 mL; nocturnal: 49 ± 26 mL) than controls (24 h: 36 ± 11 mL; nocturnal: 63 ± 24 mL).	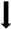	Not reported	7
Crasson et al. (2004) [59]	14 MDD (7 M, 7 F; mean age: 52 ± 8)	14 controls (6 M, 8 F; mean age M:59 ± 8, F:46 ± 5)	Not reported	HDRS: 33.5 ± 5.5	All patients underwent at least 2 week of washout period	MLT measured via blood (over 15 h period) and urine (over 24 h period).	Between-groups	A significant delayed MLT peak (77 min later) in patients (0330 h ± 23 min) than controls (0213 h ± 25 min).	*~*	Not reported	7
Carvalho et al. (2006) [63]	Phase 1: 32 MDD (9 M, 23 F; mean age: 33.6 ± 1.6)	Phase 1: 32 controls (9 M, 23 F; mean age: 33.2 ± 1.7)	Not reported	MDD: HDRS: 17.7 ± 0.9 Controls: HDRS: 0.5 ± 0.2	All patients drug-free for at least 2 weeks	MLT levels (over 24 h period) measured via urine sample.	Between- groups	No significant differences in MLT between patients and controls in phase 1 (patients: 53.50 ± 4.60; controls: 55.60 ± 7.40) or phase 2 (patients: 41.60 ± 6.90, controls: 51.80 ± 4.50).	=	MLT did not correlate with age but relationship between MLT and symptom rating and medication not reported.	7
	Phase 2: 15 MDD patients (5 M, 10 F; mean age: 35.9 ± 1.9)	Phase 2: 15 controls (5 M, 10 F; mean age: 35.0 ± 2.2)		MDD: HDRS: 18.0 ± 1.2 Controls: HDRS: 0.5 ± 0.2							
Buckley & Schatzberg (2010) [57]	6 MDD (2 M, 4 F; mean age: 42.17 ± 11.79)	6 controls (2 M, 4 F; mean age: 35.5 ± 12.65)	Not reported	HDRS: 24.5 ± 2.07	Not reported	MLT (over 18 h period) measured via blood.	Between- groups	Significantly lower MLT in patients (22.67 ± 9.08 mL) relative to controls (47.81 ± 14.76 mL).	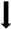	Not reported	6
Khalegipour et al. (2012) [58]	42 MDD (14 M, 28 F; mean age: 37.83 ± 7.70)	50 controls (24 M, 26 F; mean age: 36.64 ± 6.82)	Not reported	Patients: BDI-II: 27.57 ± 7.15 Controls: BDI-II: 2.46 ± 2.38	One week prior to melatonin assessment, patients asked to not take any medication. (Medication doses/type and mean age of medication duration not reported)	MLT (over 16 h period) measured via blood.	Between-groups	Significantly lower nocturnal MLT level in patients (41.27 ± 10.29 mL) relative to controls (67.42 ± 16.17 mL).	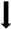	No significant sex differences in either group for morning MLT levels, but females had significantly reduced nocturnal MLT level in both patients and controls. Relationship between MLT and symptom ratings not reported.	6
Bumb et al. (2016) [65]	44 UD (19 M, 25 F; mean age: 33 ± 9.7) 37 BD (24 M, 13 F; mean age: 34.4 ± 11.8)	27 controls (11 M, 16 F; mean age: 29.1 ± 8.2)	Not reported	UD: HDRS: 18.5 ± 7 BD: YMRS: 17.8 ± 11.4	UD: 6 patients receiving antipsychotics, 24 antidepressants, and 21 receiving benzodiazepines. BD: 16 patients receiving antipsychotics, 4 antidepressants, 12 benzodiazepines and 4 receiving lithium.	MLT (between 8 a.m. and 10 a.m.) measured via CSF and blood.	Between-groups	Reduced CSF (8.5 ± 2.9 mL) (but not serum; 11.4 ± 5.2 mL) MLT in BD patients and reduced serum (9.7 ± 5.6 mL) (but not CSF; 9.1 ± 2.9 mL) in depressed patients, relative to controls (CSF:10.6 ± 7.5 mL; serum:10.4 ± 3.5 mL). No correlation was found between CSF and serum MLT levels.	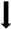	CSF and serum MLT levels did not correlate with age, symptom ratings (HAMD) or medication but correlated positively with BMI.	7
Parry et al. (2019) [60]	9 antepartum women with MDD (mean age: 28.1 ± 6.2)	17 F controls (mean age: 24.9 ± 4.1)	Antepartum AOI not reported DoI 26.3 ± 9.1 (weeks)	Baseline Antepartum patients: HDRS: 13.6 ± 4.1 (range:8–22); SIGH-ADS: 25.5 ± 6.3 (range:18–34) Healthy controls: HDRS: 4 ± 1.8 (range:1–7); SIGH-ADS: 8.9 ± 4 (range = 2–12)	All participants drug free	MLT (over 17 h period) measured via blood.	Cross-over The effects of early-night wake therapy (sleep 3 a.m. to 7 a.m.) and late-night wake therapy (sleep 9 p.m. to 1 a.m.) therapies (cross-over) in the antepartum and postpartum groups.	The baseline time from MLT onset to sleep onset approximately 2 h longer in antepartum patients than healthy controls. Early-night wake therapy realigned melatonin rhythm and increased sleep timing in antepartum depressed group. The postpartum group did not show melatonin rhythm abnormalities.	*~*	Not reported	9
	16 postpartum women with MDD (mean age: 31.0 ± 5.7)	8 F controls (mean age: 29.6 ± 6.1)	Postpartum AOI not reported DoI 18.2 ± 12.8 (weeks)	Baseline Postpartum patients: HDRS: 15.3 ± 6.1 (range:6–29); SIGH-ADS: 27.6 ± 8.5 (range:14–42) Healthy controls: HDRS: 1.5 ± 1.3 (range:0–4); SIGH-ADS: 3.8 ± 3.7 (range = 1–10)							

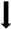
 denotes significantly lower MLT in patients compared to the healthy controls;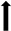 denotes higher MLT in patients relative to the healthy controls; ~denotes altered MLT secretion pattern in patients relative to healthy controls; = denotes no difference between the patient and the healthy control group in MLT level or secretion pattern. Abbreviations: AIO, Age at Illness Onset; BDI-II, Beck Depression Inventory Second Edition [75]; BMI, Body Mass Index; CPRS, Comprehensive Psychopathological Rating Scale [76]; CRG, Carney Roth Garside Questionnaire [77]; CSF, Cerebrospinal Fluid; DoI, Duration of Illness; HDRS, Hamilton Depression Rating Scale [36]; MLT, Melatonin; SZ, Schizophrenia; YMRS, Young Mania Rating Scale [78].

**Table 4:** Melatonin production in people with schizophrenia compared to those with depressive disorders and health controls.

Author & Year (in Chronological Order)	Patients (*n*, Diagnosis, Sex and Age)	Healthy Comparison Group (*n*, Sex and Age)	Patient Characteristics			Technique Used to Measure MLT	Study Design	Key Outcomes			Study Quality Assessment (NOS Total) Scores
			Age at Illness Onset (AIO) and Duration of Illness (DoI)	Symptom Rating Measure/s (Mean ± SD)	Treatment History and Medication Duration			Group Differences	Direction of Group Effects	Association with Patient Characteristics	
Steiner et al. (1990) [67]	12 SZ (sex not reported; mean age: 35.3 ± 16.1)	Controls from an earlier study [79] utilised for comparison. Controls (not detailed)	Not reported	HDRS: 19.3 ± 14.9	Participants were not given medications 8 days prior and throughout the study. (Medication doses/type and mean age of medication duration not reported).	MLT (over a 21 h period) measured via blood.	Between and within analysis	Nocturnal MLT was significantly higher in SZ patients (57.2 ± 29.1 mL) than in MDD/DST+ (45.5 ± 32.1 mL), MDD/DST- (45.2 ± 27.9 mL) and IDD (29.2 ± 17.2) at 2 a.m. and also at 11 p.m. (SZ:45.6 ± 25.0 mL; MDD/DST+:30.0 ± 26.7 mL; MDD/DST-:37.9 ± 28.6 mL; IDD:18.5 ± 7.5 mL). Nocturnal MLT in SZ patients was similar to that reported in the healthy group by Arato and colleagues (1984).	SZ < MDD SZ = Healthy Controls MDD = IDD	MLT did not correlate with demographics (sex, height, weight, height -weight ratio) except for age (r: −0.31) MLT did not correlate with depression (HDRS) ratings.	4
	49 MDD (sex not reported; mean age: 45.7 ± 14.5) 11 IDD (sex not reported; mean age: 39.4 ± 13.1)	Controls (not included)		MDD: HDRS: 22.9 ± 10.3 IDD: HDRS: 17.4 ± 7.5				MLT did not significantly differentiate the MDD/DST+, MDD/DST-, IDD groups. Only at 23:00 h MLT was significantly greater in MDD patients with normal dexamethasone suppression than MDD with dexamethasone suppression or IDD (see above for values)			

Abbreviations: AIO, Age at Illness Onset; DoI, Duration of Illness; IDD, Intermittent Depressive Disorder; MLT, Melatonin; HDRS, Hamilton Depression Rating Scale [36]; DST+, Dexamethasone Suppression Test Non-suppressor; DST-, Dexamethasone Suppression Test Suppressor; SZ, Schizophrenia.

**Table 5:** Studies examining medication output on melatonin production in patients with mood disorders compared to controls.

Author & Year (in Chronological Order)	Patients (*n*, Diagnosis, Sex and Age)	Healthy Comparison Group (*n*, Sex and Age)	Patient Characteristics		Study Design	Intervention & Procedure	Technique Used to Measure MLT	Key Outcomes		Critical Appraisal Skills Programme (CASP) Total Scores
			Age of onset (AIO) and Duration of illness (DoI)	Symptom Rating				Group & Medication Effect in MLT	Association with Symptoms	
Kennedy & Brown (1992) [68]	15 MDD patients (all F; 9 on desipramine, mean age: 38.2 ± 9.6 and 6 on adinazolam, mean age: 37.8 ± 13.6)	13 controls (all F; mean age: 25.2 ± 4.6)	Not reported	Patient on desipramine: HDRS: Week 0: 25.4 ± 5.2 Week 1: 19.2 ± 4.2 Week 3: 19.8 ± 8.2 Week 6: 18.8 ± 9.2 Patient on adinazolam: HDRS: Week 0: 23.7 ± 5.2 Week 1: 16.8 ± 4.9 Week 3: 17.3 ± 6.6 Week 6: 14 ± 8.1 Controls: HDRS: 2.3 ± 2.4	Longitudinal (6-week trial)	All patients drug free for at least 2 weeks at study entry, and received desipramine (*n* = 9) or adinazolam (*n* = 6) during the study	6-sulphatoxy MLT (over 24 h period at baseline, week 1, 3, 6) active drug therapy via urine.	At baseline, no significant difference was observed in 24 h 6-sulphatoxy MLT between patient (6.3 ± 3.4 mmoles/24 h) and controls (6.6 ± 0.8 mmoles/24 h).	MLT did not correlate with depression scores in controls and adinazolam-treated group, but a negative correlation (r = −0.068) between MLT and depression in desipramine-treated group after week 3.	11 (Funding information not available)
								Patients taking desipramine showed significant MLT increase after week 1 (10.2 ± 4.9 mmoles/24 h) relative to baseline (6.6 ± 2.9 mmoles/24 h) but it decreased to baseline by week 6 (7.0 ± 2.2 mmoles/24 h).		
Childs et al. (1995) [69]	10 seasonal affective disorder patients (1 M, 9 F; mean age: 40 ± 2.9)	10 controls (1 M, 9 F; mean age: 39.3 ± 3.0)	Not reported	HDRS: Baseline: 37.3 ± 2.4 Week 1: 25.5 ± 1.4 Week 6: 20.6 ± 3.3	Longitudinal (6-week trial)	All patients were drugs free for at least 2 weeks at study entry; then received fluoxetine (20 mg/day) during the study	MLT (over 24 h period) measured via blood.	MLT did not differ significantly between patients and controls at any stage. In patients, significant MLT reduction at week 1 of treatment, relative to baseline, observed at 2:00 a.m., 2:30 a.m., and 3:00 a.m. This effect disappeared by week 6. (mean values not reported).	Relationship between MLT, symptom severity and demographics not reported.	10 (Funded by Wessex medical trust and Eli Lilly limited)
Szymanska et al. (2001) [70]	20 major depression patients (5 M, 15 F; mean age: 45.9 ± 8.4)	14 controls (8 M, 6 F; mean age: 41.5 ± 4.0)	AIO Not reported; DoI 2–5 months	HDRS, BDI (mean values not reported).	Longitudinal (8-week trial)	14 patients stopped antidepressants and underwent a 7-day washout period and 6 were medication free for 1+ year before receiving the clomipramine.	MLT (over 24 h period) measured via blood.	Mean MLT was significantly higher in patients (1494.4 ± 159.1 mL/24 h) than controls (986.7 ± 71.8 mL/24 h) at midnight, 2 a.m., and 4 a.m. No significant differences were observed in MLT levels from pre- to post- clomipramine treatment.	MLT did not correlate with symptom severity (HARDS and BDI). No significant difference in MLT found between patients with severe and moderate depression levels.	10 (Funding information not available)
Tan et al. (2007) [71]	13 MDD patients (7 M, 6 F; mean age: 26.85 ± 4.71)	13 controls (sex distribution not reported; mean age: 26.92 ± 4.13)	Not reported	Patients: HDRS: Week 0: 20.08 ± 2.91 Week 1: 13.5 ± 2.51 Week 2: 10.75 ± 3.37 Week 4: 8.13 ± 4.16 Week 6: 5.25 ± 3.33 SDS: Week 0: 53 ± 7.21 Week 1: 46.75 ± 5.92 Week 2: 42.12 ± 4.94 Week 4: 37.5 ± 5.58 Week 6: 33.75 ± 5.52	Longitudinal (4-week trial)	All patients given fluoxetine (20 mg) during the study; and 5 patients took estazolam or lorazepam during the first 2 weeks (if required).	MLT (over 24 h period) measured via saliva.	No significant difference in salivary peak MLT levels of patients (pre-treatment: 86.82 ± 35.72; post-treatment: 42.13 ± 24.43) and controls (68.18 ± 28.08). No significant difference between pre- and post- fluoxetine treatment MLT levels	MLT did not correlate with symptom severity.	10 (Funded by Nature science foundation)

Abbreviations: AIO, Age at Illness Onset; BDI, Beck’s Depression Inventory [32]; DoI, Duration of Illness; HDRS, Hamilton Depression Rating Scale [36]; MDD, Major Depressive Disorder; POMS, Profile of Mood States [74]; SDS, Zung Self-Rating Depression Scale [80]; MLT, Melatonin.

## 4. Discussion

This systematic review evaluated currently existing evidence to identify possible aberrations in pineal gland (PG) volume and melatonin production in people with psychosis or mood disorders and their possible associations with patient characteristics, especially symptom severity.

### 4.1. PG Volume in Psychosis and Mood Disorders

Our review of existing findings in psychosis and mood disorders yielded consistent evidence of smaller PG volumes in first-episode and chronic schizophrenia patients as well as in people at a high risk of developing psychosis, compared to healthy controls, with no significant influence of symptom severity or medication status [16,17,28,29], and also some evidence of smaller PG volumes in schizophrenia patients, relative to mood disorder patients [16]. The same direction of effects (i.e., smaller PG volume in patients than controls) was present in people with bipolar disorders [16] but not significantly so in all studies [30,31]. Smaller-than-normal PG volume was also present in people with major depression, independent of symptom severity [16,18], although there was also evidence of this being applicable to only certain sub-types, or to those experiencing ‘loss of interest’ as a symptom [30]. Taken together, these findings suggest that smaller-than-normal PG volume may represent a transdiagnostic biomarker for schizophrenia and mood disorders, deserving of further study in relation to sex differences, functional and clinical outcomes, including treatment responsiveness.

### 4.2. MLT Production and Secretion Patterns in Psychosis and Mood Disorders

The majority of the existing studies conducted in schizophrenia observed lower-than-normal MLT in acutely unwell as well in chronic patient groups [45,46,47,48] or an atypical pattern of MLT secretion in this clinical population [49,50,51]. The studies, however, also showed some differences between acutely ill and chronic patients [48] and there was cross-sectional evidence of possible normalisation of MLT secretion pattern in medicated patients [51], suggesting that MLT production might be influenced by both illness and medication-related factors. As yet, there are no published longitudinal investigations of such possible influences in MLT production of schizophrenia and related populations.

Our review of studies in people with major depression revealed considerable evidence of lower-than-normal MLT, especially nocturnal MLT [49,52,53,54,55,56,57], or atypical MLT secretion patterns, including delayed peak [59] or delayed MLT onset [60]. However, this was not found in some studies [62,63] and appeared in others to be dependent on presence of psychotic symptoms [64] or methods for assessing MLT [66]. Some data showed an association between lower MLT and greater depression severity [53]. The findings in bipolar disorder patients were also mixed, again suggesting that MLT in major depression and bipolar disorders may be sensitive to multiple influences. Lastly, there was some evidence of a transient lowering of MLT following the initiation of certain antidepressants in drug-free patients [desipramine [68]; fluoxetine [69]] suggesting that medication may have been a confounding factor and contributed to the mixed pattern of MLT findings in mood disorders. Interestingly, all four studies that investigated medication effects on MLT showed either no MLT difference between the patient and control groups (3 studies), or higher MLT in depressed patients relative to controls (one study), at baseline. This is hard to explain and may relate to some non-specific patient selection bias in such studies (e.g., selection of only those patients who were considered safe enough to withdraw medication for some time in order to meet study eligibility criteria).)

### 4.3. Limitations of the Reviewed Evidence and the Review Processes

The findings of this systematic review should be considered taking a number of limitations into account. First, most of the reviewed studies had small-to-moderate sample sizes (with *n*< 50 per group), providing limited power to compare PG structure and function between the patient and healthy control groups and to investigate sex differences in diagnosis-related effects; therefore, they are likely to have been underpowered to meaningfully examine any associations between the PG structure/function and patient characteristics of potential relevance, especially symptom profiles. Second, a number of studies either did not provide sufficient information on patient characteristics or did not examine them in relation to the PG structure or function, which would have allowed greater insight into this topic and a clearer interpretation of negative results (for example, 31). Third, the methods for assessing MLT varied greatly between the reviewed studies with MLT assessed via different routes (in blood, urine, CSF) and the samples collected at different times (e.g., one-off, in the morning or evening, over 8–24 h). Fourth, there were only four studies examining antidepressant effects on MLT in mood disorders, and all of these were of fair quality at best (see Supplementary Table S2); and there were no studies assessing antipsychotic effects on the PG structure or function in people with psychosis. Making solid conclusions about diagnosis or symptom related differences without a clear understanding of medication influences in the PG structure or function is difficult. Lastly, we deliberately focused on psychosis and mood disorders but it is possible that the PG structure and function are also affected in other disorders, and did not conduct a meta-analysis to formally assess the effect of the moderator variables (due to the limited number of studies with required power and information available for review), restricting confidence in some of our findings until they are examined and supported by future research.

## 5. Conclusions and Future Directions

Our review provided consistent evidence that smaller-than-normal PG volume represents a transdiagnostic biomarker across psychosis and mood disorders, and possibly other disorders linked with sleep dysfunction. We also found lower-than-normal MLT as well as aberrant MLT secretion pattern (flattened or shifted rhythm) in psychosis and mood disorder patients, relative to healthy controls, but the findings on these measures appeared fragile, possibly due to various influencing factors, such as severity of certain symptoms, specific symptom profiles and medications. With these findings, we make a number of recommendations for the future scientific enquiry in this area.

First, we suggest that future clinical studies should employ a transdiagnostic approach involving multiple patient groups and assessment on multiple symptom dimensions, including the quality of sleep and sleep disturbances. A similar approach can be taken to clarify the association between specific symptom dimensions and PG volume and function in non-clinical samples, especially to rule out medication-related confounders and potentially identify underlying latent factors.

Second, we emphasise that future studies should have large enough sample sizes to detect diagnosis related effects of small-to-medium size. The studies should also aim to include sufficient number of males and females to allow meaningful investigation of sex differences, given their influence in many brain structures and function [81] as well as in prevalence rate, prognosis, symptom profile and presentation, and treatment response in many disorders, including schizophrenia [82,83] and affective disorders [84].

Third, we recommend future studies to consider the chronotype of study participants as well as take any regional and seasonal variations into account for both the control and patient groups, given their relevance for mental disorders as well as MLT production [85].

Fourth, we highlight the need for multimodal investigations involving assessment of both PG structure and function in the same samples to clearly understand how PG volume aberrations, with or without covarying for whole-brain volume, may influence MLT production in the short and long-term in interaction with age, BMI, sex, and possible illness- or symptom-related influences in clinical and non-clinical samples.

Finally, we encourage future studies to examine how currently used antipsychotics for treating psychosis might influence the PG volume and MLT in parallel to changes in sleep parameters, and also consider the use of melatonin as potential treatment avenues for reducing side effects of antipsychotics [86,87,88], ameliorating various cognitive and information processing disturbances typically present in people with schizophrenia [89,90], and treating sleep disturbances in a range of psychiatric disorders, including but not limited to psychosis and mood disorder [91,92].

## Data Availability

Not applicable.

## Supplementary Materials

The following supporting information can be downloaded at: https://www.mdpi.com/article/10.3390/brainsci13050827/s1, Table S1: Newcastle-Ottawa Quality Assessment Scale (NOS) scores for the selected studies., Table S2: Critical Appraisal Skills Programme (CASP) scores.
