# Supplementary material for: Pineal Abnormalities in Psychosis and Mood Disorders: A Systematic Review
Source: Brain Sci. 2023 May 20;13(5):827. doi: 10.3390/brainsci13050827 (PMC10216209; doi:10.3390/brainsci13050827)
Supplement: Supplementary file 1 [file brainsci-13-00827-s001.zip › brainsci-2364694-supplementary.pdf]

**Supplementary Materials**

**Supplementary Table S1.** Newcastle-Ottawa Quality Assessment Scale (NOS) scores for the selected studies. It has three domains namely selection, comparability, and outcome to evaluate cross-sectional studies. Each dimension is rated individually, the maximum score for *'selection'* is 3 and minimum is 0, for *'comparability'* the maximum score is 2 and minimum is 0, and for *'outcome'* the maximum is 2 and minimum is 0. For *'selection'* domain a score of 3 is good, 2 is fair, and 0-1 is poor. For *'comparability'* and *'outcome'* domains, a score of 2 is good, 1 is fair, and 0 is poor.

| **Selected studies** | **Selection** | **Comparability** | **Outcome** |
|---|---|---|---|
| Rajarethinam et al. (1985) [19] | Fair | Poor | Fair |
| Bersani et al. (2002) [28] | Good | Fair | Fair |
| Takahashi et al. (2019) [29] | Good | Fair | Good |
| Takahashi et al. (2022) [17] | Good | Fair | Fair |
| Findikli et al. (2015) [16] | Good | Good | Fair |
| Sarrazin et al. (2012) [31] | Good | Good | Fair |
| Zhao et al. (2019) [18] | Good | Good | Fair |
| Takahashi et al. (2020) [30] | Good | Good | Fair |
| Ferrier et al. (1982) [45] | Good | Good | Fair |
| Beckman et al. (1984) [44] | Good | Fair | Fair |
| Fanget et al. (1989) [46] | Good | Poor | Fair |
| Monteleone et al. (1992) [49] | Good | Good | Fair |
| Rao et al. (1994) [51] | Good | Poor | Good |
| Jiang & Wang (1998) [47] | Good | Good | Fair |
| Vigano et al. (2001) [48] | Good | Good | Fair |
| Wulff et al. (2012) [50] | Good | Good | Fair |
| Beck-Friis et al. (1984) [52] | Good | Poor | Fair |
| Claustrat et al. (1984) [54] | Good | Fair | Fair |
| Brown et al. (1985) [55] | Good | Fair | Fair |
| Beck-Friis et al. (1985) [53] | Good | Poor | Good |
| Thompson et al. (1988) [62] | Good | Good | Fair |
| McIntyre et al. (1989) [56] | Good | Poor | Fair |
| Kennedy et al. (1996) [66] | Good | Poor | Fair |
| Shafii et al. (1996) [64] | Good | Poor | Fair |
| Voderholzer et al. (1997) [61] | Good | Good | Fair |
| Crasson et al. (2004) [59] | Good | Good | Fair |
| Carvalho et al. (2006) [63] | Good | Good | Fair |
| Buckley & Schatzberg (2010) [57] | Good | Fair | Fair |
| Khalegipour et al. (2012) [58] | Good | Fair | Fair |

| | | | |
|---|---|---|---|
| Bumb et al. (2016) [65] | Good | Good | Fair |
| Parry et al. (2019) [60] | Good | Good | Good |
| Steiner et al. (1990) [67] | Fair | Poor | Fair |

**Supplementary Table S2.** Critical Appraisal Skills Programme (CASP) scores. CASP consists of four domains with 11 items evaluating randomised trials. Response to each item is recorded as 'yes', 'no', and 'can't tell'. The first three items are eliminatory; and only if the response is 'yes', the rater continues with remaining items (assessing design, methodology, results, and benefits/importance of research).

**CASP Items** (for information)

1. Did the study address a clear focused research question?
2. Was the assignment of the participants to intervention randomised?
3. Were all participants who entered the study accounted for at its conclusion?
4. Was the study methodologically sound?
5. Were the study groups similar at the start of the randomised controlled trial?
6. Apart from the experimental intervention, did each study group receive the same level of care?
7. Were the effects of the intervention reported comprehensively?
8. Was the precision of the estimate of the intervention or treatment effect reported?
9. Do the benefits of the experimental intervention outweigh the harms and costs?
10. Can the results be applied to your local population/in your context?
11. Would the experimental intervention provide greater value to the people in your care than any of the existing intervention?

*Each item scored after reading selected papers. A score of 2 was given for 'yes', 1 for 'no', and 0 for 'cannot tell'.*

**CASP Ratings for Selected Studies**

| Study | Item 1 | Item 2 | Item 3 | Item 4 | Item 5 | Item 6 | Item 7 | Item 8 | Item 9 | Item 10 | Item 11 | Funding Source |
|---|---|---|---|---|---|---|---|---|---|---|---|---|
| Kennedy & Brown (1992) [68] | 2 | 0 | 2 | 0 | 1 | 2 | 2 | 0 | 0 | 2 | 0 | Funder Not reported |
| Childs et al. (1995) [69] | 2 | 0 | 2 | 0 | 1 | 2 | 1 | 0 | 0 | 2 | 0 | Wessex medical trust and Eli Lilly limited |
| Szymanska et al. (2001) [70] | 2 | 0 | 2 | 0 | 1 | 2 | 1 | 0 | 0 | 2 | 0 | Funder Not reported |
| Tan et al. (2007) [71] | 2 | 0 | 2 | 0 | 1 | 1 | 2 | 0 | 0 | 2 | 0 | Nature science foundation |
